# Predicting Outcomes for Interhospital Transferred Patients of Emergency General Surgery

**DOI:** 10.1155/2022/8137735

**Published:** 2022-04-15

**Authors:** Brandon Cave, Daniel Najafali, William Gilliam, Jackson F. Barr, Christian Cain, Chris Yum, Jamie Palmer, Safura Tanveer, Emily Esposito, Quincy K. Tran

**Affiliations:** ^1^University of Maryland School of Medicine, Baltimore, MD, USA; ^2^The Research Associate Program in Emergency Medicine and Critical Care, Department of Emergency Medicine, University of Maryland School of Medicine, Baltimore, MD, USA; ^3^Carle Illinois College of Medicine, University of Illinois Urbana-Champaign, Urbana, IL, USA; ^4^The R Adams Cowley Shock Trauma Center, University of Maryland School of Medicine, Baltimore, MD, USA; ^5^Department of Emergency Medicine, University of Maryland School of Medicine, Baltimore, MD, USA

## Abstract

**Background:**

Interhospital transferred (IHT) emergency general surgery (EGS) patients are associated with high care intensity and mortality. However, prior studies do not focus on patient-level data. Our study, using each IHT patient's data, aimed to understand the underlying cause for IHT EGS patients' outcomes. We hypothesized that transfer origin of EGS patients impacts outcomes due to critical illness as indicated by higher Sequential Organ Failure Assessment (SOFA) score and disease severity.

**Materials and Methods:**

We conducted a retrospective analysis of all adult patients transferred to our quaternary academic center's EGS service from 01/2014 to 12/2016. Only patients transferred to our hospital with EGS service as the primary service were eligible. We used multivariable logistic regression and probit analysis to measure the association of patients' clinical factors and their outcomes (mortality and survivors' hospital length of stay [HLOS]).

**Results:**

We analyzed 708 patients, 280 (39%) from an ICU, 175 (25%) from an ED, and 253 (36%) from a surgical ward. Compared to ED patients, patients transferred from the ICU had higher mean (SD) SOFA score (5.7 (4.5) vs. 2.39 (2), *P* < 0.001), longer HLOS, and higher mortality. Transferring from ICU (OR 2.95, 95% CI 1.36–6.41, *P*=0.006), requiring laparotomy (OR 1.96, 95% CI 1.04–3.70, *P*=0.039), and SOFA score (OR 1.22, 95% CI 1.13–1.32, *P* < 0.001) were associated with higher mortality.

**Conclusions:**

At our academic center, patients transferred from an ICU were more critically ill and had longer HLOS and higher mortality. We identified SOFA score and a few conditions and diagnoses as associated with patients' outcomes. Further studies are needed to confirm our observation.

## 1. Introduction

Emergency general surgery (EGS) is a field that has substantial growth with 2.7 million admissions per year between 2001 and 2010 [1, 2]. Coupled with the shifting paradigm of regionalized medicine, there has been an increase in interhospital transfers (IHT, transfers between different hospitals) among EGS patients [3]. These patients are initially admitted to general surgery at a local hospital or admitted to a local hospital until a surgical problem is found and cared for until their disease severity exceeds the capabilities of the index facility [4]. Once the complexity of care surpasses the resources available and the level of care provided is insufficient, these patients are transferred to tertiary hospitals with comprehensive specialty coverage and specialized medical teams, such as a dedicated EGS service. Previous literature established that these EGS patients who are transferred are associated with higher rates of mortality and require higher care intensity at accepting facilities; however, patient-level analysis has not been conducted to quantify the degree of injury and disease severity of the patients who are being transferred [5,6]. Moreover, patients' clinical features and factors specific to the initial hospital prior to admission to EGS and their contributions to their increased mortality have not been fully elucidated.

The increased mortality and higher care requirements of EGS patients arriving from IHT are associated with increased cost and resource utilization [5]. While interhospital EGS transfers have risen annually, there has been a reduction in the surgical workforce responsible for treating them [7]. The available literature has analyzed patients from an epidemiological perspective using aggregate scoring mechanisms to stratify their patient populations [1, 3, 5, 7]. Despite this, analysis has not been completed that accounts for the origin of transfers among this patient population once arriving at transferring facilities that associate these patients with worse outcomes and increased resource utilization, such as an evaluation of EGS patients from the emergency department (ED), intermediate care unit (IMC), or intensive care unit (ICU). Each of these locations have different disease severities and risk of poor outcome. Thus, the cohorts in prior studies have been limited because of grouping patients together into a single transfer population. Additionally, the nature of emergency general surgery is the urgency of the disease processes necessitating prompt medical management and surgical intervention if necessary. The corresponding EGS service is resource intensive and requires multiple teams working in tandem to facilitate optimal care. Dedicated acute care surgery services accommodate emergent procedures more rapidly and help improve patient outcomes.

Patients that are transferred to a tertiary hospital for higher levels of care have worse outcomes than those who are received through the ED [5, 8]. Our approach stratified patients into categories based on location prior to IHT in order to more equitably appreciate the differences that transferring a patient can have on their outcome. We aimed to understand the differences in disease severity among EGS patients who were transferred to a tertiary care center. Our study utilized our hospital's large patient population transferred from other hospitals to our center's quaternary care EGS service. We analyzed patient-level data, such as laboratory values, Sequential Organ Failure Assessment (SOFA) scoring, and care provided to identify predictors of mortality, hospital length of stay (HLOS), and discharge disposition. Our study aim was to provide a patient-level analysis to help referring facilities and accepting physicians during the decision-making process when transferring a patient to a higher level of care. We hypothesized that interhospital transferred EGS patients from inpatient units are more critically ill than patients who were transferred directly from the EDs. Understanding these patients' clinical features will improve patients' overall care once transferred and under the care of an EGS service.

## 2. Methods

### 2.1. Study Setting and Patient Selection

Our data was retrospectively collected from patients who were admitted to our academic quaternary medical center's EGS service from January 1, 2014, to December 31, 2016. Our institution's EGS service is available around-the-clock and accepts patients from our own ED and throughout the entire state and surrounding region. The goal of this service is to evaluate the need for emergent surgery and to expedite the management of patients with urgent general surgical conditions that exceed the capabilities of the referring hospitals. The EGS service at our institution is composed of an EGS attending physician, an EGS fellow, and senior and junior general surgery residents. The EGS team evaluates the patients and formulates a management plan as soon as the patients arrive at our institution. Admitted patients are cared for directly by our EGS team. Patients who are admitted to the surgical intensive care unit (SICU) benefit from multidisciplinary and collaborative care teams composed of the EGS team and intensive care providers.

Our study population included all adult patients who were transferred to our hospital with EGS service as the primary service. These patients were transferred from other hospitals' EDs, ICUs, or any inpatient units (intermediate care unit (IMC), stepdown unit, or regular surgical ward). We excluded patients who were not admitted to EGS as the primary service. For example, we excluded patients who were accepted by vascular surgery or gynecology specialties but with EGS serving as a consulting service. Additionally, due to our institution's dedicated trauma center and accompanied trauma service, EGS does not see a high volume of trauma patients. We excluded patients with incomplete medical records. This study was approved by our institutional review board (IRB).

### 2.2. Primary and Secondary Outcomes

Our primary outcome of interest was in-hospital mortality. We further characterized mortality according to the referring locations (ED, ICU, or inpatient units) as independent variables. Our secondary outcomes were patients' hospital dispositions and survivors' HLOS. Patients who died during hospitalization were excluded from our HLOS analysis.

### 2.3. Data Collection and Management

Data was collected retrospectively from our institution's electronic health records (EHR). The principal investigator first trained the research team members to extract data from sets of 10 patient charts until interrater's agreement with a senior research team member's results reached at least 90%. An experienced research team member randomly checked an additional 10% of the data during the data extraction phase. To further reduce the risk of bias, research team members also extracted data in separate sections. For example, investigators who extracted laboratory data did not have access to outcome variables. Data was entered into a standardized Microsoft Access database (Microsoft Corp., Seattle, WA, USA). Data was subsequently deidentified prior to analysis.

We extracted patients' demographic information, including age, race, sex, and comorbidities. The extracted laboratory values upon admission at our institution included serum lactate level, white blood cell (WBC) count, and components of the SOFA score. We imputed the missing SOFA components as normal values. Furthermore, as serum lactate levels are not usually checked among patients who are admitted to our surgical ward, we categorized patients who were admitted to our surgical ward without serum lactate laboratory values as having normoperfusion, which we defined as serum lactate level ≤2 millimoles per liter (mmol/L).

We also collected data about patients' interventions, such as any pretransfer surgical operations. From our own institution, we collected the estimated blood loss (EBL) volume and the amount of crystalloid administration during the first surgical operations. Finally, we collected primary diagnosis upon discharge and each patient's hospital discharge disposition.

### 2.4. Sample Size Calculation

We planned to perform multivariable logistic regression to measure the association between clinical independent variables and mortality. To estimate the sample size for our multivariable logistic regressions, we used the following formula [9].

(1) $$\begin{array}{c} \text{sample size}=\frac{\left(\text{number of counts per independent variables}\right)*\left(\text{number of independent variables}\right)}{\left(\text{incidence of outcome}\right)}. \end{array}$$

We planned to have a multivariable logistic regression that supports 10 independent variables with five events per independent variable [10], assuming the incidence of mortality as 8% as previously reported [7]. As a result, we needed approximately 600 patients for our multivariable logistic regression to support 10 independent variables.

### 2.5. Statistical Analysis

We presented continuous variables that are normally distributed with mean and standard deviation (SD). We used median and interquartile range (IQR) when summarizing nonparametric data. Analysis of continuous data was carried out using either a Student *t*-test or Mann–Whitney *U* test when appropriate. Categorical variables were reported as *N* (%) and compared using either a Pearson *χ*^2^ test or a Fisher exact test when appropriate.

To measure the associations between demographic and clinical independent variables and mortality, we used forward stepwise multivariable logistic regression to avoid overfitting our model. For our multivariable logistic regression, we reported results as odds ratio (OR), 95% confidence interval (95% CI), and the *P*-value. We evaluated any potential collinearity that could have been present in variables that comprised our regression models using variance inflation factor (VIF). All independent variables in our regression had a VIF <5 and had minimal interaction with other terms; thus, they were deemed appropriate for analysis. The corresponding VIF are reported as appropriate. We used the Hosmer–Lemeshow test to evaluate the goodness of fit of our multivariable logistic regression. A model with a Hosmer–Lemeshow *P* > 0.05 was considered a good fit.

For the secondary outcomes of hospital dispositions and survivors' HLOS, we used ordinal logistic regressions. We ranked the hospital dispositions from the lowest order as (1) discharge to home, (2) discharge to a rehabilitation or subacute facility, (3) discharge to a skilled nursing facility, and (4) discharge to hospice or death. Similarly, we ranked the order of survivors' HLOS from the lowest order of (1) short stay to (2) medium and (3) long stay. Prior to ranking HLOS order, we examined the histogram of patients' HLOS and ranked the HLOS from ≤5 days (short) to 5 to 12 days (medium) and >12 days (long). We reported the results from the ordinal logistic regression as correlation coefficient (corr. coeff.), 95% CI, and *P*-value. An independent variable with positive correlation coefficient would be associated with the lowest rank of the ordinal regression and the next one after it. An independent variable with negative correlation coefficient would be associated with the highest rank of the ordinal regression and the next one below it. The design of the Minitab (version 19) statistical software does not retain any information regarding nonsignificant variables in the stepwise multivariable logistic regressions. As a result, we cannot present the statistical information for these nonsignificant independent variables from our regressions. In order to report all variables chosen in all our regression models, we presented them for each model in Table 1.

### 2.6. Additional Analysis

We also performed univariate probit logit analyses to examine the probability of mortality with continuous independent variables that were identified by the multivariable logistic regression as significantly associated with mortality. We also used the area under the receiver operating characteristic curve (AUROC) analysis to assess the discriminatory capabilities of our probit logit analyses. An AUROC of 1 showed perfect discriminatory capability between dead (coded as 1) and alive (coded as 0), while an AUROC of 0.5 showed poor discrimination between the dichotomous outcomes.

Data analysis was conducted using Minitab version 19 (https://www.minitab.com; Minitab LLC, State College, PA, USA). Analyses with two-tailed *P* < 0.05 were considered statistically significant.

## 3. Results

### 3.1. Characteristics of EGS Patients

**Figure 1:**
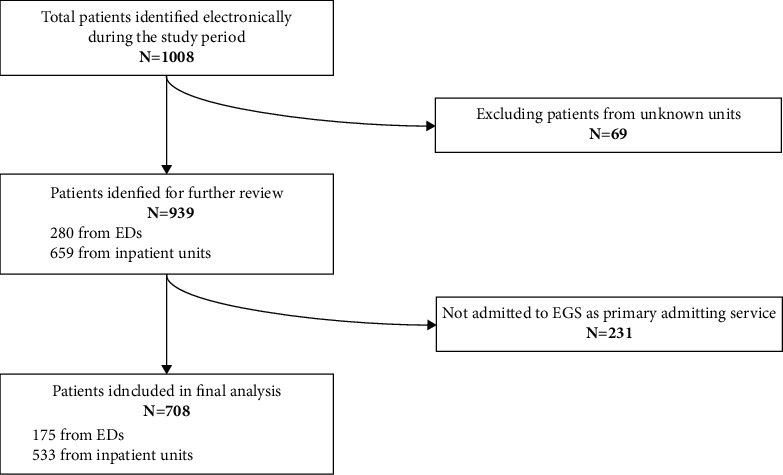
Patient selection diagram mapping out patients who were transferred for EGS and included in the final analysis. ED: emergency department; EGS: emergency general surgery.

We electronically identified 1008 patients during the study period. A total of 300 patients were excluded from the study which was made up of 69 patients who were excluded because their transferring unit was unknown and an additional 231 patients were excluded because EGS was not the primary admitting service. After further chart review, we identified and included 708 patients who were transferred to our academic center's EGS, as primary service, from other hospitals (Figure 1). There were 175 patients (25%) who were admitted from other hospitals ED; the mean age (±SD) was 61 (17) years for this group. There were 280 patients (39%) who were transferred from other hospitals' ICUs and whose mean age was 60 (16) years. Two hundred fifty-three (253, 36%) patients were transferred from inpatient units and this group's mean age was 56 (16) years (Table 2).

The most frequent diagnosis among patients admitted from the ED was bowel obstruction (15%), whereas patients from the ICU predominantly had intra-abdominal infections (17%). Both patients from the ED and from the ICU had laparotomy as the most frequent procedure, but patients from inpatient units predominantly received laparoscopic procedures. Patients from other inpatient units had higher rates of nonoperative management (64%) compared to the ED (28%) and ICU (34%), respectively.

Overall mortality for patients transferred for EGS in our study population was 9% (*N* = 61). Among our entire cohort, 51 (18%) patients from the ICU had an open abdomen upon admission to our EGS service. Patients from the ICU had a significantly higher mean (SD) SOFA score when compared to patients from the ED (5.70 (4.50) vs. 2.39 (2.18), *P* < 0.001), but when compared to patients from other inpatient units, the ED had significantly higher mean (SD) SOFA score (2.39 (2.18) vs. 1.40 (2.26), *P* < 0.001) (Table 3). Compared to patients transferred from the ED, patients from the ICU had higher mean WBC counts (in thousands of cells per microliter of blood (K/mcL)) and higher serum lactate (in millimoles per liter (mmol/l)), while patients from inpatient units had lower WBC and serum lactate (Table 3). EBL in milliliters (mL) in the index surgical operation at upon arrival at our institution was statistically similar when comparing the ED and the ICU (*P*=0.05) and the ED and the other inpatient units (*P*=0.54).

### 3.2. Primary Outcome: Mortality after Transfer of EGS Patients

Patients who were transferred from the ICU had highest hospital mortality, while patients from ED or inpatient units had similar rates of mortality (Table 3).

Forward stepwise multivariable regression analysis (Table 4) was performed to investigate the association between patients' clinical factors and mortality. Each incremental point in a patient's total SOFA score was significantly associated with a 22% increased likelihood of mortality (OR 1.22, 95% CI 1.13–1.32, *P* < 0.001). Other factors associated with increased risk of mortality were increased age (OR 1.07, 95% CI 1.05–1.10, *P* < 0.001), undergoing an emergent laparotomy (OR 1.96, 95% CI 1.04–3.70, *P*=0.039), and being admitted to the EGS service from the ICU (OR 2.95, 95% CI 1.36–6.41, *P*=0.006). Additionally, gastrointestinal (GI) bleeding and a past medical history (PMHx) of diabetes mellitus (DM) were associated with an 80% (OR 0.20, 95% CI 0.05–0.79, *P*=0.021) and 54% (OR 0.46, 95% CI 0.22–0.96, *P*=0.038) lower chance of death after transfer, respectively. The model showed good fit of the independent variable as its Hosmer–Lemeshow goodness-of-fit test returned a *P* > 0.05.

### 3.3. Secondary Outcomes: HLOS and Hospital Disposition

For our secondary outcomes', HLOS and patient's discharge disposition, associations with clinical factors were measured using two separate ordinal logistic regressions (Table 5). Hospital survivors who were transferred from the ICU (14 (8–25) days) had higher median (IQR) HLOS compared to both patients who were transferred from ED (7 (4–11) days, *P* < 0.001) or inpatient units (6 (3–9) days) (Table 3). Patients who were transferred from the ED also had higher HLOS than patients who were transferred from inpatient units. Factors associated with patients' increased likelihood for short HLOS (≤5 days) were a diagnosis of appendicitis (corr. coeff. +2.08, 95% CI 1.41–45.81, *P*=0.019), diagnosis of liver or gallbladder infection (corr. coeff. +0.94, 95% CI 1.32–5.01, *P*=0.006), and admission from the ED (corr. coeff. +0.46, 95% CI 1.07–2.33, *P*=0.021). In contrast, having a fistula (corr. coeff. −1.01, 95% CI 0.17–0.77, *P*=0.009), undergoing a laparotomy (corr. coeff. −0.83, 95% CI 0.26–0.72, *P*=0.001), each incremental increase in a patient's total SOFA score (corr. coeff. −0.20, 95% CI 0.77–0.87, *P* < 0.001), and hypoperfusion based on serum lactate levels (corr. coeff. −0.59, 95% CI 0.35–0.88, *P*=0.011) were associated with long HLOS (>12 days). This model also showed good fit of independent variables as its deviance test, goodness-of-fit test, returned *P* > 0.05 for this analysis.

Patients who were originally admitted from the ED (group A) had a significantly higher rate of being discharged home (127, 72%), when compared to the ICU (group B) (102, 36%, *P* < 0.001), but their rate of being discharged home was statistically similar to patients who were transferred from other inpatient units (group C) (172, 68%, *P*=0.31). One factor significantly associated with discharge home was admission from the ED (corr. coeff. +0.63, 95% CI 1.13–3.09, *P*=0.015). Three diagnoses (bowel perforation, fistula, and intra-abdominal infection) and undergoing laparotomy were significantly associated with hospice or mortality. Additional factors significantly associated with hospice or mortality were increased age (corr. coeff. −0.06, 95% CI 0.93–0.95, *P* < 0.001), each additional increment in a patient's total SOFA score, and having hypoperfusion based on serum lactate levels. This model's Deviance Test, goodness-of-fit test, returned a *P* > 0.05 for this analysis, suggesting a good fit of the independent variables.

### 3.4. Probit Analysis: Mortality

**Figure 2:**
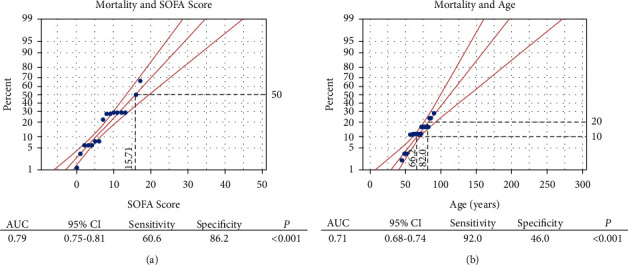
Probit and AUROC analysis for EGS patients' outcome of mortality. AUC: area under the ROC curve; CI: confidence interval; EGS: emergency general surgery; ROC: receiver operating characteristic; and SOFA: Sequential Organ Failure Assessment.

Probit analysis was used to investigate the probability of hospital death with certain levels of patients' continuous clinical variables (age and total SOFA score), after they were identified by our multivariable logistic regression. Univariate probit analysis found that both patients' total SOFA score (*P* < 0.001) and age (*P* < 0.001) were significantly associated with mortality. We observed that a total SOFA score of around 16 was associated with mortality in 50% of patients (Figure 2(a)). Similarly, probit analysis demonstrated that the likelihood of mortality increases with advancing age (Figure 2(b)).

## 4. Discussion

Our study demonstrated differences in demographics, clinical factors, and outcomes between patients who were transferred from the ED and inpatient settings to our EGS service. We identified clinical factors that were associated with patient mortality, HLOS, and hospital discharge disposition. Disease severity, as measured through SOFA scoring at the time of transfer, had a significant association with patient outcomes.

From our study's results, we believe that immediate assessment and SOFA score calculation should be used when determining the risk severity of an IHT patient. The clinical criteria contained within the SOFA scoring system is typically available as part of the standard of care for inpatient units on arrival to the transfer facility, which can be utilized proactively to guide patient management. Physicians should look to design novel algorithms and investigate available scoring mechanisms, like SOFA, that can help predict risk following transfer should we continue a healthcare model that regionalizes care and concentrates subspecialties in urban environments. IHT patients originating from an ICU constitute a substantial portion of transferred EGS patients who experience poor clinical outcomes; our study showed 17% mortality, compared to 5% and 1% for ED and non-ICU transfers, respectively. Using scoring criteria for clinical data that is already available, in the ICU particularly, may be a means to reduce mortality in the ICU IHT population. We found that patients with diabetes and patients with gastrointestinal bleeding were associated with lower mortality. Our study used patients' self-report of their past medical history such as diabetes and we did not objectively measure their hemoglobin A1C. According to Gopalan et al., many patients with diabetes are unaware of their actual hemoglobin A1c (HbA1c) status which makes it difficult to know their true glycemic severity [11]. Therefore, our study's past medical history of diagnosis may not reflect patients' comorbidities. Additionally, patients with upper gastrointestinal bleeding were found to have a mortality rate of 10% and patients with lower gastrointestinal bleeding were found to have a low all-cause in-hospital mortality rate of ∼4% [12, 13]. Therefore, our study aligns with previous investigations that found gastrointestinal bleeding to be associated with decreased risk of mortality, when compared with other disease states.

Our study design accounted for origin of transfer, which we found to have a significant impact on mortality. This finding is consistent with previously published study findings that IHT is associated with a greater risk of mortality [5, 14]. However, current available literature overlooks transfer origin as a variable in mortality risk. We quantified that patients from the ICU are a different patient population than those transferred from other inpatient units. Thus, collective EGS patients should be evaluated based on the severity of their illness beyond their admission diagnosis using clinical or physiologic criteria, like SOFA score. The ICU patients were at greater risk of morbidity and mortality and had significantly higher SOFA scores when compared with our ED group. Patients transferred from non-ICUs did not have the same patient profile as those from ICU transfer.

Therefore, when assessing the needs of a patient in the ICU, transferring them to a higher-level center before they exceed the needs of the hospital may lead to more favorable outcomes. Our institution developed an efficient system, in conjunction with the State of Maryland, to identify and facilitate transfer of these patients with time sensitive disease and help improve their outcomes [14,15]. We found additional clinical characteristics attributable to a higher mortality in the ICU transfer group. A portion (18%) of these patients were transferred with an open abdomen upon arrival to our quaternary academic center. This was not the case in patients from the ED group and non-ICU units. Patients arriving with an open abdomen require more resources and higher care intensity, which may not be available at referring hospitals [5, 15, 16]. Increased resource utilization and care requirements can contribute to poor outcomes as disease progression may outpace care provided throughout treatment.

Our data showed that patients with a higher admission SOFA score were associated with a longer HLOS. Additionally, we found certain diagnoses correlating with longer HLOS. From these data points, we show that these patients who are transferred from an ICU had significantly higher disease severity when they were transferred. Future studies may help elucidate indicators that identify disease progress, which should facilitate transfer earlier to prevent worse outcomes for transferred patients. Furthermore, future studies should investigate the relationship associated with worse outcomes and ICU transferred patients based on the timing of transfer initiation. This information is crucial to further understand the implications of transferring a critically ill patient and will allow for the development of a predictive algorithm that may be utilized to determine which patients would benefit from transfer.

### 4.1. Limitations

Our study had several limitations. Retrospective data collection prevented us from being able to account for surgical interventions received prior to arrival and other interventions critically ill patients may have received during transport. Our data included if an operation was performed prior to transfer but did not encompass the specific details of the procedure if an operation took place because of limitations in electronic health record across many different healthcare systems. Additionally, we do not have a true admission group from our academic center's ED to serve as a control group for our study. However, we had a large number of patients who came from other hospitals' EDs throughout the entire state to serve as a surrogate control group for patients from an inpatient unit.

Laboratory data was also limited with regard to nonacute levels of care that did not order lactate levels for these patients. Our mean lactate levels may not reflect the true lactate levels of these patients because many of them who were admitted to non-ICU levels of care did not have their lactate checked. Moreover, almost all patients did not have repeat lactate levels, which prevented us from evaluating lactate clearance. In order to overcome this limitation, we dichotomized patients into two groups based on their degree of hypoperfusion. This piece of analysis serves as a marker to determine those patients who were critically ill with organ dysfunction. We also did not have access to patients' hemoglobin A1c (HbA1c) to confirm our patients' diabetes status.

Our study involved patients at a quaternary care center with a specialized EGS service. This is not reflective of a standard EGS service. Our setting may present a different population of patients, which may not be generalizable to EGS as a subspecialty. Additionally, this study was conducted at a single institution, which is in a densely populated state with a high transfer volume. This may not be representative of the Western United States that has rural catchments often extending across multiple states. We have a free-standing, level-one trauma center that is also available to filter EGS patients depending on their mechanism of injury, which skews our EGS service population. This may have impacted our study populations, which could explain some differences in our study when compared to previous studies, such as our large population of patients transferred with an open abdomen. Additionally, gastroenterology is not an admitting service at our institution; therefore, patients who have either upper or lower gastrointestinal bleeds will need our EGS service to accept them so that they can be transferred to our institution. This practice may not be generalizable to other institutions.

Despite the aforementioned limitations of our study, we find our results to be applicable to other settings because our institution has a region-wide transferring process from other hospitals' inpatient units and EDs to serve as a representation of the general population [17, 18]. We also describe the characteristics and outcomes of these patients according to their referring locations, which has been a major limitation of previous studies looking at IHT patients undergoing EGS. By providing information regarding the location and patient-level data for these EGS patients, we provide insights about clinical factors as well as the level of acuity each patient received.

## 5. Conclusion

Among the rapidly growing EGS population, our study aimed to understand the differences in disease severity between patients who were transferred to a tertiary care center for EGS. Similar to other studies, we found that patients from ICUs are significantly more critically than those transferred from other units. We believe that the SOFA score is a crucial metric that physicians can use to help determine the risk of disease progression and patient mortality when deciding on patient transfer for EGS. Further work is needed to confirm our observation and to identify additional risk factors for mortality in order to improve our care of this critically ill patient population.

## Figures and Tables

**Table 1:** List of all variables included in regression models.

Variables	Classification
Forward selection multivariable logistic regression—mortality	
Age—each year	Cont.
Sex—male	Cat.
PMHx—CHF	Cat.
PMHx—dialysis	Cat.
PMHx—any kidney disease	Cat.
PMHx—any liver disease	Cat.
PMHx—DM	Cat.
PMHx—HTN	Cat.
Diagnosis—appendicitis	Cat.
Diagnosis—bowel ischemia	Cat.
Diagnosis—bowel obstruction	Cat.
Diagnosis—bowel perforation	Cat.
Diagnosis—fistula	Cat.
Diagnosis—GI bleeding	Cat.
Diagnosis—hernia	Cat.
Diagnosis—intra-abdominal infection	Cat.
Diagnosis—liver or gallbladder infection	Cat.
Diagnosis—pancreatitis	Cat.
Procedure type—endoscopy	Cat.
Procedure type—I&D	Cat.
Procedure type—laparoscopic	Cat.
Procedure type—laparotomy	Cat.
Procedure type—percutaneous intervention by IR	Cat.
Open abdomen—yes	Cat.
Total SOFA score—each point	Cont.
Lactate hypoperfusion—yes	Cat.
EBL—each mL	Cont.
Fluid in OR—each mL	Cont.
Unit—transferred from ED	Cat.
Unit—transferred from ICU	Cat.
Unit—transferred from other inpatient units	Cat.

Both multivariable ordinal logistic regressions—HLOS + disposition	
Age—each year	Cont.
Sex—male	Cat.
PMHx—CHF	Cat.
PMHx—dialysis	Cat.
PMHx—any kidney disease	Cat.
PMHx—any liver disease	Cat.
PMHx—DM	Cat.
PMHx—HTN	Cat.
Diagnosis—appendicitis	Cat.
Diagnosis—bowel ischemia	Cat.
Diagnosis—bowel obstruction	Cat.
Diagnosis—bowel perforation	Cat.
Diagnosis—fistula	Cat.
Diagnosis—GI bleeding	Cat.
Diagnosis—hernia	Cat.
Diagnosis—intra-abdominal infection	Cat.
Diagnosis—liver or gallbladder infection	Cat.
Diagnosis—pancreatitis	Cat.
Procedure type—endoscopy	Cat.
Procedure type—I&D	Cat.
Procedure type—laparoscopic	Cat.
Procedure type—laparotomy	Cat.
Procedure type—percutaneous intervention by IR	Cat.
Open abdomen—yes	Cat.
Total SOFA score—each point	Cont.
Lactate hypoperfusion—yes	Cat.
EBL—each mL	Cont.
Fluid in OR—each mL	Cont.
Unit—transferred from ED	Cat.

Cat.: categorical variable; CHF: congestive heart failure; Cont.: continuous variable; DM: diabetes mellitus; ED: emergency department; EBL: estimated blood loss; GI: gastrointestinal; HLOS: hospital length of stay; HTN: hypertension; I&D: incision and drainage; ICU: intensive care unit; IR: interventional radiology; mL: milliliter; OR: operating room; PMHx: past medical history; and SOFA: Sequential Organ Failure Assessment.

**Table 2:** Demographic and clinical characteristics of patients admitted to EGS from the ED and inpatient units.

Variables	From ED (A)	From inpatient units		*P* ^*∗*^	*P* ^*∗∗*^
		From ICU (B)	From other inpatient units (C)		
Total patients, *N*	175	280	253	NA	NA
Age (years), mean (SD)	61 (17)	60 (16)	56 (16)	0.48	**0.002**

Gender, *N* (%)
Male	86 (49)	159 (57)	111 (44)	0.11	0.28
Female	89 (51)	121 (43)	142 (56)		

PMHx, *N* (%)
HTN	38 (22)	176 (63)	112 (44)	**<0.001**	**<0.001**
DM	47 (27)	80 (29)	61 (24)	0.69	0.52
CHF	14 (8)	18 (6)	19 (8)	0.52	0.85
Any liver disease	4 (2)	43 (15)	28 (11)	**<0.001**	**0.001**
Any kidney disease	17 (10)	24 (9)	17 (7)	0.68	0.26

Diagnoses, *N* (%)
Appendicitis	6 (3)	3 (1)	1 (1)	0.09	**0.020**
Bowel obstruction	26 (15)	24 (9)	31 (12)	**0.037**	0.44
Bowel perforation	4 (2)	26 (9)	9 (4)	**0.003**	0.45
Bowel ischemia	14 (8)	17 (6)	2 (1)	0.43	**<0.001**
GI bleeding	20 (11)	33 (12)	11 (4)	0.91	**0.005**
Fistula	5 (3)	16 (6)	20 (8)	0.16	**0.029**
Hernia	6 (3)	6 (2)	16 (6)	0.55	0.18
Intra-abdominal infection	23 (13)	47 (17)	34 (13)	0.30	0.93
Liver or gallbladder infection	5 (3)	22 (8)	32 (13)	**0.028**	**<0.001**
Pancreatitis	22 (13)	42 (15)	39 (15)	0.47	0.41
Perforated viscera	25 (14)	NA	NA	NA	NA
Other	19 (11)	43 (15)	58 (23)	0.17	**0.001**

Operation, *N* (%)
None	49 (28)	94 (34)	162 (64)	0.21	**<0.001**
Laparotomy	52 (30)	129 (46)	33 (13)	**0.001**	**<0.001**
Laparoscopy	11 (6)	13 (5)	38 (15)	0.45	**0.005**
Endoscopy	20 (11)	1 (1)	1 (1)	**<0.001**	**<0.001**
Percutaneous intervention by IR	10 (6)	10 (4)	2 (1)	0.28	**0.005**
I&D	8 (5)	4 (1)	6 (2)	0.07	0.21
Other	25 (14)	29 (9)	11 (4)	0.21	**<0.001**
Any operation prior to transfer, *N* (%)	NA	81 (29)	28 (11)	NA	NA

^*∗*^Statistical analysis of Group A (patients from ED) versus Group B (patients from ICU). ^*∗∗*^Statistical analysis of Group A (patients from ED) versus Group C (patients from other inpatient units). Bold cells indicate statistically significant variables (*P* < 0.05). CHF: congestive heart failure; DM: diabetes mellitus; ED: emergency department; EGS: emergency general surgery; GI: gastrointestinal; HTN: hypertension; I&D: incision and drainage; ICU: intensive care unit; IR: interventional radiology; NA: not applicable; PMHx: past medical history; and SD: standard deviation.

**Table 3:** Clinical features of patients admitted to EGS from the ED and inpatient units.

Variables		From ED (A)	From inpatient units		*P* ^*∗*^	*P* ^*∗∗*^
			From ICU (B)	From other inpatient units (C)		
Total patients, *N*		175	280	253	NA	NA
SOFA score, mean (SD)^¶^		2.39 (2.18)	5.70 (4.50)	1.40 (2.26)	**<0.001**	**<0.001**
WBC count (K/mcL), mean (SD)		11.74 (6.60)	15.00 (9.08)	9.83 (5.15)	**<0.001**	**<0.001**
Serum lactate (mmol/L), mean (SD)		2.00 (1.51)	2.50 (2.73)	1.57 (1.08)	**0.016**	**0.022**
Patients with open abdomen, *N* (%)		NA	51 (18)	NA	NA	NA
EBL of first operation (mL), mean (SD)		143.73 (752.66)	271.00 (851.65)	91.66 (311.81)	0.05	0.54
Fluid in operating room (mL), mean (SD)		718.53 (1351.37)	2035.16 (2936.39)	917.00 (1764.45)	**<0.001**	0.86

Blood products during first operation^#^, *N* (%)						
pRBCs	15 (9)		76 (27)	19 (8)	**<0.001**	0.69
FFP	9 (5)		43 (15)	7 (3)	**0.001**	0.20
Platelets	3 (2)		18 (6)	18 (7)	**0.020**	**0.011**
Other blood products	26 (15)		61 (22)	20 (8)	0.07	**0.022**
Survivors' HLOS (days), median [IQR]^§^	7 [4–11]		14 [8–25]	6 [3–9]	**<0.001**	**0.020**
Patients with short hospital stay^##^, *N* (%)	68 (41)		29 (12)	116 (46)	**<0.001**	0.25
Patients with medium hospital stay^##^, *N* (%)	61 (37)		71 (31)	94 (38)	0.22	0.82
Patients with long hospital stay^##^, *N* (%)	38 (22)		132 (57)	40 (16)	**<0.001**	0.08

Type of disposition, *N* (%)						
Discharge home	127 (72)		102 (36)	172 (68)	**<0.001**	0.31
Rehab or subacute facility	30 (17)		94 (34)	43 (17)	**<0.001**	0.97
Skilled nursing facility	10 (6)		15 (5)	7 (3)	0.87	0.13
Hospice or expired	8 (5)		48 (17)	3 (1)	**<0.001**	0.06

^*∗*^Statistical analysis of Group A (patients from ED) versus Group B (patients from ICU). ^*∗∗*^Statistical analysis of Group A (patients from ED) versus Group C (patients from other inpatient units). ^¶^Higher score indicates more severe disease. ^§^Total patients (*N* = 649) after removing 59 patients who died prior to discharge, from ED = 167, from ICU = 232, and from inpatient units = 250. ^#^Denotes the total blood products administered to the entire patient population, not the number of blood products per patient. ^##^Patients' HLOS was categorized based on the following criteria: Short HLOS: ≤5 days. Medium HLOS: >5 days to ≤12 days. Long HLOS: >12 days. Bold cells indicate statistically significant variables (*P* < 0.05). ED: emergency department; EGS: emergency general surgery; EBL: estimated blood loss; FFP: fresh frozen plasma; HLOS: hospital length of stay; ICU: intensive care unit; IQR: interquartile range; mL: milliliters; mmol/L: millimoles per liter; NA: not applicable; pRBCs: packed red blood cells; SOFA: Sequential Organ Failure Assessment; SD: standard deviation; K/mcL: thousands of cells per microliter of blood; and WBC: white blood cell.

**Table 4:** Multivariable logistic regression using forward selection with *α* = 0.10 measuring association of clinical factors with mortality. Only variables that were statistically significant were reported.

Variables	Multivariable regression			
	OR	95% CI	*P*	VIF
Primary outcome: Mortality^†^				
Unit—ICU	2.95	1.36–6.41	0.006	1.36
Procedure type—laparotomy	1.96	1.04–3.70	0.039	1.12
Total SOFA score—each point^¶^	1.22	1.13–1.32	<0.001	1.40
Age—each year	1.07	1.05–1.10	<0.001	1.17
PMHx—DM	0.46	0.22–0.96	0.038	1.03
Diagnosis—GI bleeding	0.20	0.05–0.79	0.021	1.05

^¶^Higher score indicates more severe disease. Goodness-of-fit test: ^†^Hosmer–Lemeshow Test. Degrees of freedom: 8, *χ*^2^: 2.82, *P*=0.95. CI: confidence interval; DM: diabetes mellitus; GI: gastrointestinal; ICU: intensive care unit; OR: odds ratio; PMHx: past medical history; SOFA: Sequential Organ Failure Assessment; and VIF: variance inflation factor.

**Table 5:** Multivariable ordinal logistic regression measuring association of clinical factors with patients' outcomes of HLOS and disposition. The order for HLOS was 0 (short, ≤5 days), 1 (medium, >5 days to ≤12 days), and 2 (long, >12 days). The order for disposition was 1 (home), 2 (rehab or subacute facility), 3 (skilled nursing facility), and 4 (discharge to hospice or death). All independent variables selected a priori were included in the models; only variables that were statistically significant were reported.

Variables	Ordinal logistic regression		
	Coefficient	95% CI	*P*
Outcome: HLOS^†^			
Diagnosis—appendicitis	2.08	1.41–45.81	0.019
Diagnosis—liver or gallbladder infection	0.94	1.32–5.01	0.006
Unit—transferred from ED	0.46	1.07–2.33	0.021
Diagnosis—fistula	−1.01	0.17–0.77	0.009
Procedure type—laparotomy	−0.83	0.26–0.72	0.001
Lactate hypoperfusion	−0.59	0.35–0.88	0.011
Total SOFA score—each point^¶^	−0.20	0.77–0.87	<0.001

Outcome: disposition^‡^			
Unit—transferred from ED	0.63	1.13–3.09	0.015
Diagnosis—fistula	−1.82	0.05–0.53	0.003
Diagnosis—bowel perforation	−1.44	0.07–0.77	0.017
Diagnosis—intra-abdominal infection	−1.34	0.09–0.77	0.015
Procedure type—laparotomy	−0.71	0.30–0.81	0.006
Lactate hypoperfusion	-0.61	0.35–0.83	0.005
Total SOFA score—each point^¶^	−0.15	0.82–0.90	<0.001
Age—each year	−0.06	0.93–0.95	<0.001

^¶^Higher score indicates more severe disease. Goodness-of-fit test: ^†^Deviance test. Degrees of freedom: 1267, *χ*^2^: 1159, *P*=0.99. ^**‡**^Deviance test; Degrees of freedom: 2091, *χ*^2^: 1117, *P* = 0.99. (+) correlation coefficient is associated with the lowest rank of the ordinal regression and the next one after it; (+) value is associated with lower HLOS and discharge home. (−) correlation coefficient is associated with the highest rank of the ordinal regression and the next one below it; (−) value is associated with longer HLOS and discharge to hospice or death. CI: confidence interval; ED: emergency department; HLOS: hospital length of stay; OR: odds ratio; SOFA: Sequential Organ Failure Assessment.

## Data Availability

Due to the agreement with our IRB at our institution, we cannot share the data presented in this manuscript.
